# Supplementary material for: Development of FGF21 Mutant with Potent Cardioprotective Effects in T2D Mice via FGFR1–AMPK-Mediated Inhibition of Oxidative Stress
Source: Int J Mol Sci. 2025 Jul 9;26(14):6577. doi: 10.3390/ijms26146577 (PMC12295313; doi:10.3390/ijms26146577)
Supplement: Supplementary file 1 [file ijms-26-06577-s001.zip › ijms-3671991-supplementary.pdf]

SUPPLEMENTARY INFORMATION

Development of FGF21 Mutant with Potent Cardioprotective Effects in T2D Mice via FGFR1–AMPK-Mediated Inhibition of Oxidative Stress

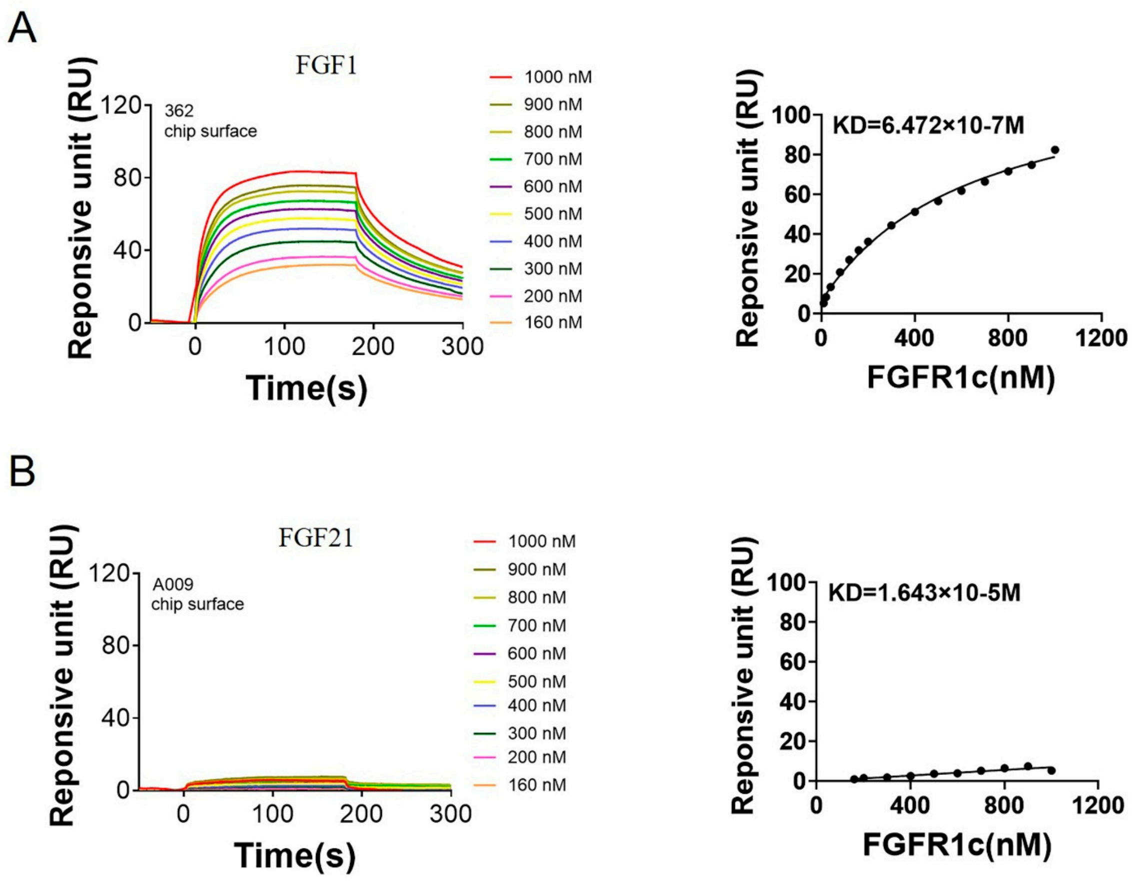

Figure S1. FGF1 has a higher biding affinity than FGF21 toward FGFR1c.

(A, B) Representative SPR sensorgrams of binding interactions of FGF1 **(A)** and $FGF21^{WT}$ **(B)** with the extracellular ligand-binding domain of FGFR1c (left hand side) and the saturation binding curves used to derive equilibrium dissociation constants (K_d) (right hand side). All data were expressed as mean ± SEM.
